# An *operando* spatially resolved study of alkaline battery discharge using a novel hyperspectral detector and X-ray tomography

**DOI:** 10.1107/S1600576720012078

**Published:** 2020-10-13

**Authors:** Thomas Connolley, Oxana V. Magdysyuk, Stefan Michalik, Phoebe K. Allan, Manuela Klaus, Paul H. Kamm, Francisco Garcia-Moreno, Jennifer A. Nelson, Matthew C. Veale, Matthew D. Wilson

**Affiliations:** a Diamond Light Source Ltd, Harwell Campus, Didcot, Oxfordshire OX11 0DE, United Kingdom; b School of Chemistry, University of Birmingham, Haworth Building, Edgbaston, Birmingham B15 2TT, United Kingdom; c Helmholtz-Zentrum Berlin für Materialien und Energie, Hahn-Meitner-Platz 1, Berlin, 14109, Germany; d Duracell Inc., Bethel, Connecticut, USA; e Rutherford Appleton Laboratory, Science and Technology Facilities Council, Harwell Campus, Didcot, Oxfordshire OX11 0QX, United Kingdom

**Keywords:** hyperspectral detector, AA batteries, X-ray diffraction, X-ray tomography

## Abstract

An experimental technique is described for the collection of time-resolved X-ray diffraction information from a complete commercial battery cell during discharging or charging cycles. The technique uses an 80 × 80 pixel 2D energy-discriminating detector in a pinhole camera geometry which can be used with a polychromatic X-ray source. The concept was proved in a synchrotron X-ray study of commercial alkaline Zn–MnO_2_ AA size cells. Importantly, no modification of the cell was required. The technique enabled spatial and temporal changes to be observed with a time resolution of 20 min (5 min of data collection with a 15 min wait between scans). Chemical changes in the cell determined from diffraction information were correlated with complementary X-ray tomography scans performed on similar cells from the same batch. The clearest results were for the spatial and temporal changes in the Zn anode. Spatially, there was a sequential transformation of Zn to ZnO in the direction from the separator towards the current collector. Temporally, it was possible to track the transformation of Zn to ZnO during the discharge and follow the corresponding changes in the cathode.

## Abstract 2

An experimental setup is described that uses a hyperspectral imaging detector to collect time-resolved X-ray diffraction information from a complete discharging AA size battery, using a commercial alkaline Zn–Mn cell as a proof of concept. The work is complemented by time-resolved *in situ* X-ray computed tomography of an identical battery cell.

## 1. Introduction   

The increase in renewable energy generation requires more efficient batteries for energy storage, resulting in a greater demand for development of *in situ* and *operando* techniques for battery characterization during various charge/discharge processes under different environmental conditions. *In situ* techniques provide a deeper understanding of the working mechanisms for prototype and new batteries. Making measurements without opening the batteries is important to avoid exposing the battery contents to air, which can affect the observed results.

Specialist cells for *operando* characterization have been constructed, for example using optical microscopy to study damage which may occur during deep discharge of alkaline Zn–MnO_2_ cells (Faegh *et al.*, 2018 ▸). X-ray methods, especially at synchrotron light sources, permit *in situ operando* studies of standard battery designs such as coin cells or AA cells with metal outer casings which are opaque to other techniques. The use of energy-dispersive X-ray diffraction (EDXD) for characterization of materials within operating batteries has recently been reviewed (Marschilok *et al.*, 2020 ▸). Using slits on the incident and diffracted beams to define a gauge volume within a sample means that EDXD is spatially resolved, typically in the range of tens to hundreds of micrometres. The high X-ray flux at a synchrotron source enables sufficiently fast data collection for time-resolved study of charging, discharging or cycling of batteries. The spatial resolution of EDXD has been used to compare the structure of electrochemical phases in the cathode in charged and discharged prototype LiMn_2_O_4_-based CR2032 coin cells (Liang *et al.*, 2013 ▸). Strobridge *et al.* (2015 ▸) used time-resolved EDXD to map the evolution of the inhomogeneous electrochemical reaction in an experimental coin cell with a carbon-coated LiFePO_4_ cathode. Li/Ag_2_VP_2_O_8_ coin cells were used by Kirshenbaum *et al.* (2015 ▸) to study the effect of discharge rate on battery capacity. *In situ* EDXD, complemented by *ex situ* X-ray absorption spectroscopy, identified a rate-dependent discharge mechanism, and showed that lower discharge rates and higher battery capacity correlated with a more even distribution of Ag atoms. Other *in situ* EDXD work has been done on Zn/MnO_2_ alkaline batteries (Gallaway *et al.*, 2014 ▸, 2015 ▸; Bhadra *et al.*, 2015 ▸). The motivation for studying alkaline batteries was their potential for use in rechargeable systems for energy storage. Monitoring of chemical changes during cyclic charging and discharging of LR20 (D cell) batteries revealed precipitation of ZnO near the separator in the anode, which can be a cause of failure after a high number of cycles (Gallaway *et al.*, 2014 ▸). Work on intact alkaline AA cells discharged at different rates was able to distinguish the discharge products in the MnO_2_ cathode (Gallaway *et al.*, 2015 ▸). An intriguing experiment, supported by synchrotron EDXD measurements, correlated changes in the coefficient of restitution of an alkaline AA cell in a ‘bounce test’ with the formation of ZnO in the anode (Bhadra *et al.*, 2015 ▸).

X-ray diffraction, which is sensitive to crystallographic (and hence chemical) changes, can be complemented by radiographic and tomographic imaging techniques which are used to study microstructural and morphological changes such as cracking, collapse or gas pocket formation (Finegan *et al.*, 2015 ▸, 2017 ▸). Zinc–air batteries have been studied *in situ* using tomography (Schröder *et al.*, 2014 ▸; Arlt *et al.*, 2014 ▸; Franke-Lang *et al.*, 2017 ▸). An *in situ* discharge study used tomography to monitor LR61 AAAA alkaline cells, revealing spatial changes in the anode over time (Haibel *et al.*, 2010 ▸), and *in situ* tomography has recently been used to study morphological changes in rechargeable alkaline Zn/MnO_2_ cells (Osenberg *et al.*, 2018 ▸).

Established techniques like X-ray diffraction and tomography are complemented by the recent introduction of hyperspectral instruments, such as the high-energy X-ray imaging technology (HEXITEC) detector. The HEXITEC detector (Seller *et al.*, 2011 ▸; Jones *et al.*, 2009 ▸; Veale *et al.*, 2018 ▸) is an 80 × 80 pixel CdTe instrument with 250 µm pixels, capable of simultaneous imaging and spectroscopy. It is a hyperspectral detector, meaning that each pixel collects a complete photon energy spectrum up to 200 keV. The average FWHM energy resolution is 0.75 keV for incident photons of 59.54 keV, and 1.1 keV at 141 keV (Veale *et al.*, 2012 ▸). The detector can be used as a straight radiographic instrument, or to collect fluorescence and/or X-ray scattering signals, with laboratory or synchrotron X-ray sources. Jacques *et al.* (2013 ▸) demonstrated the use of HEXITEC in radiographic mode with a laboratory source, showing how plastic, metal and ceramic components of an object could be identified from the spectral information in each pixel. In fluorescence mode, 2D maps of several elements were obtained simultaneously, and a pinhole was used to improve spatial resolution. The pinhole technique was then applied by Egan and co-workers to white-beam energy-dispersive diffraction imaging (also known as dark-field imaging) at a synchrotron source (Egan *et al.*, 2014 ▸) and with a laboratory source (Egan *et al.*, 2015 ▸). In their work, 2D dark-field imaging was applied to study the microstructure of an aluminium friction stir weld, revealing differences in texture which were not apparent in a conventional radiograph. The high flux of synchrotron X-ray sources opens up the possibility of time-resolved hyperspectral imaging, as demonstrated by Liotti *et al.* (2015 ▸), who used a HEXITEC detector in fluorescence geometry to map multiple elements during melting and solidification of metallic samples. Another application where time and spatial resolution is valuable is in the study of batteries, which provided the motivation for the work that will be described in this paper. The HEXITEC detector has the advantage of giving 2D information in each image, rather than having to raster the battery sample through a small X-ray beam to build up a map, which is done in the EDXD technique.

We describe two complementary experiments, the first using energy-dispersive diffraction with a 2D HEXITEC detector, and the second using conventional monochromatic absorption contrast tomography. The experiments were conducted on commercially available Zn/MnO_2_ alkaline AA cells (Duracell), in order to monitor the chemical and structural changes taking place during battery discharge. We chose to work with alkaline cells because of their well known chemistry. Primarily, this work was conducted to prove the concept of using a HEXITEC detector for an *operando* study of a complete battery system. The diffraction technique, together with the complementary tomography method, offers the ability to conduct engineering-level studies of complete battery systems under realistic operating conditions, without having to perform interrupted tests or destructive sectioning of batteries at different time steps.

## 2. Experimental methods   

### 2.1. Selection of battery and region of interest   

**Figure 1:**
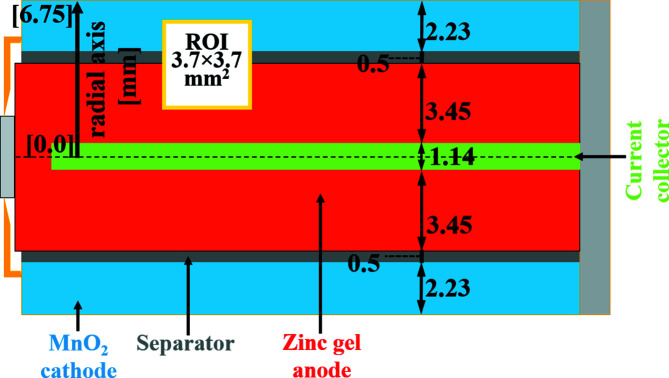
A simplified cross section of the investigated alkaline AA battery, with indicated dimensions along the radial axis in millimetres and showing the region of interest (ROI) for the *operando* study with the HEXITEC hyperspectral detector during battery discharge.

The battery chosen for this study was an alkaline AA size cell based on Zn–MnO_2_ chemistry. This is a ubiquitous high-energy-density battery for consumer applications. There is interest in using the same chemical system for large-scale energy storage, because the materials are safe and inexpensive (Gallaway *et al.*, 2014 ▸, 2015 ▸; Ingale *et al.*, 2015 ▸; Turney *et al.*, 2017 ▸). A cross section through an AA Zn–MnO_2_ cell is shown schematically in Fig. 1 ▸. The anode consists of zinc particles, which transform to ZnO during battery discharge. The cathode dis MnO_2_, which transforms to manganese oxy­hydroxide, MnOOH, during battery discharge; the electrolyte is potassium hydroxide. There are known to be other reaction products such as Mn_3_O_4_ (hausmannite) and ZnMn_2_O_4_ (hetaerolite), the formation of which could affect battery performance (Gallaway *et al.*, 2015 ▸, 2016 ▸). The chemical equations for the reactions occurring during battery discharge are as follows:

(i) In the anode, hydroxide ions react with Zn to form zincate ions according to the electrochemical reaction
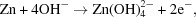
followed by the precipitation of ZnO,
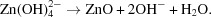



(ii) In the cathode, MnO_2_ is reduced to MnOOH:
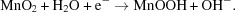



Overall, oxidation of Zn to zincate Zn(OH)_4_^2−^ releases two electrons, which flow through the external circuit powered by the battery to the cathode, where MnO_2_ is reduced to MnOOH. A further transformation of zincate to zinc oxide can occur in the anode. The formation of ZnO can limit the ability of the cell to be recharged, although the formation of ZnO in alkaline cells is not fully understood (Gallaway *et al.*, 2014 ▸).

Our selected region of interest (ROI; Fig. 1 ▸) was the anode/separator/cathode region near the top of the current collector, our aim being to characterize radial changes in both anode and cathode chemistry and physical structure over time. The advantage of the HEXITEC detector over conventional EDXD is that it is a full-field imaging technique, allowing for a whole area to be mapped in a single shot, rather than point-by-point, which has the potential to improve time resolution. In this study, we compared batteries that were continuously discharged with those that experienced interrupted discharge, to look for evidence of differences in reaction pathway or product distribution induced by pauses in discharge.

### 2.2. *Operando* spatially resolved diffraction   

The diffraction experiment was performed on the EDDI beamline at the BESSY-II synchrotron, Helmholtz Zentrum, Berlin (Klaus & Garcia-Moreno, 2016 ▸; Jiménez *et al.*, 2018 ▸). The white beam from the beamline’s 7 T multipole wiggler was filtered with 10 mm of aluminium to attenuate low-energy photons which could cause sample heating, and to harden the incident X-ray spectrum to higher photon energies. This filtration ensured that the highest-intensity diffraction peaks are above the Cd and Te self-absorption edges of the detector (26.7 and 31.8 keV, respectively). Filtration also kept the count rate at a level to be able to identify charge.

#### 2.2.1. Pinhole camera for diffraction measurements   

**Figure 2:**
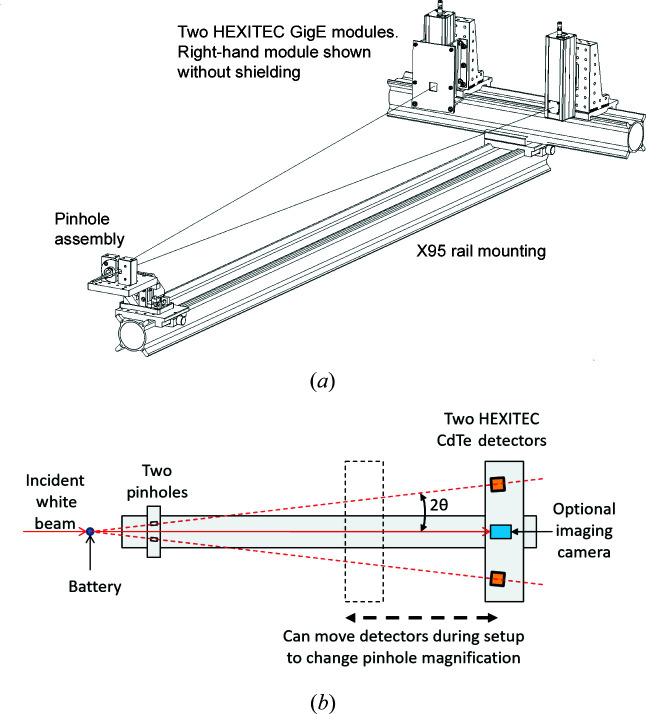
(*a*) A drawing of the dual HEXITEC detector and pinhole system, mounted on an X95 optical rail. (*b*) A schematic diagram of the experimental setup used for the *operando* battery diffraction experiments.

Spatially resolved diffraction information was recorded with a HEXITEC CdTe pixellated hyperspectral detector (Seller *et al.*, 2011 ▸; Jacques *et al.*, 2013 ▸; Veale *et al.*, 2018 ▸) using a pinhole magnification technique (Egan *et al.*, 2014 ▸, 2015 ▸) to give a field of view of 3.7 × 3.7 mm. A dual HEXITEC detector system and sample stage was assembled and tested on Beamline I12-JEEP at Diamond Light Source (Drakopoulos *et al.*, 2015 ▸), and then transported to and installed on the EDDI beamline at BESSY-II. Geometric parameters for the HEXITEC detector setup are given in Table 1 ▸. A drawing of the HEXITEC system and a schematic of the experimental setup are shown in Fig. 2 ▸. The latest development of the HEXITEC dark-field setup shown in Fig. 2 ▸ uses two detectors, one on each side of the direct beam path. It is possible to combine the measurements from both detectors to improve signal *versus* background compared with a single detector. In this paper, we present the results from one detector only.

A small X-ray imaging camera was included in the setup for direct radiographic imaging of the sample. Acquiring radiographs with the camera was a significant aid to initial sample alignment. In principle, the setup could be developed further to acquire radiographs during battery discharge experiments, but this was not done on this occasion.

#### 2.2.2. Calibration of HEXITEC detector   

Energy calibration of the HEXITEC detector was carried out prior to the experiment using sealed X-ray sources. Once set up on the beamline, per-pixel diffraction calibration was performed, because the spatial extent of the 20 × 20 mm sensor means that each pixel is at a slightly different 2θ angle with respect to the sample position. Diffraction calibration data were collected using NIST 640d Si and 660a LaB_6_ powder standards, mounted in flat 0.5 mm thick shim sample holders. Diffraction images obtained from the flat shim calibration samples were then used to correlate the energy spectrum bins in each pixel with known lattice parameters.

Firstly, for each pixel, Si diffraction peaks 111, 220 and 311 were identified and then fitted by a Gaussian function to get Si peak position values *E_hkl_* in an energy domain. The profile fitting of the Gaussian function was implemented through the MATLAB function *lsqcurvefit*, which solves nonlinear least-squares problems (MATLAB 2012 version, MathWorks Inc.). *E_hkl_* values were directly associated with values of the momentum transfer vector *Q_hkl_* through the equation *Q* = 4π*E*sin(θ)/(*hc*), where *h* and *c* are Planck’s constant and the speed of light, respectively. Then pair points (*E*_111_, *Q*_111_), (*E*_220_, *Q*_220_) and (*E*_311_, *Q*_311_) were linearly fitted using the MATLAB function *polyfit* to get coefficients *p*(1) and *p*(2). Finally, knowing the parameters of the linear dependence between the momentum transfer vector and energy for all the pixels, the *Q* value of each bin of a particular pixel was calculated. To control the performed procedure of *Q*-axis calibration, the collected LaB_6_ data were inspected. The positions of the LaB_6_ diffraction peaks were found to be in accordance with expected values within ±0.01 Å^−1^.

#### 2.2.3. Limitations of pinhole camera technique   

In performing this feasibility study of a pinhole camera for diffraction measurements, some limitations of the technique were encountered. First, the limitations intrinsic to any pinhole camera will be discussed. The image formed by a pinhole camera is a convolution of the source object with the point spread function (PSF) of the pinhole and the PSF of the imaging sensor (Thomas *et al.*, 2010 ▸; Elleaume *et al.*, 1995 ▸). The PSF of the pinhole has a diffraction contribution and a geometric contribution due to the finite size of the pinhole. The diffraction contribution increases as the pinhole diameter decreases, while the geometric contribution increases with increasing pinhole diameter. Using the simple analytical approach provided by Thomas *et al.* (2010 ▸), the optimum pinhole size at 40 keV for the HEXITEC setup used for the battery experiment is approximately 5 µm, which is similar to another analytical solution given by Harrison *et al.* (2014 ▸). Apart from being difficult to manufacture, such a small pinhole would result in very long data collection times. Instead, a large 200 µm diameter pinhole was used to ensure sufficient signal in the 5 min collection time we wanted to achieve. At this size, the geometric contribution dominates. Again using a simple analytical approach (Thomas *et al.*, 2010 ▸), and assuming a Gaussian source, the root-mean-square (r.m.s.) size of the pinhole’s contribution to the PSF is 372 µm. The PSF of the HEXITEC sensor has been modelled and measured (Koch-Mehrin *et al.*, 2020 ▸), with the conclusion that the average charge cloud size is fairly constant from 5 to 100 keV with an r.m.s. width of 13 µm, which is well below the 250 µm pixel size. Noting that the r.m.s. width of a Gaussian is the standard deviation, ±3 standard deviations means that ∼99.7% of charge would be collected in a diameter of 78 µm, which is still within one pixel. With contributions to the overall PSF added in quadrature (Thomas *et al.*, 2010 ▸), the overall system PSF r.m.s. width due to contributions from the pinhole and sensor is 372.3 µm or 1.5 pixels, dominated by the geometric contribution of the pinhole aperture. The outcome is that, although the magnification of the pinhole camera gives a pixel resolution of 46 µm per pixel (Table 1 ▸), the standard deviation on this is 1.5 pixels or 69 µm, and there will be overlap in signal between pixels due to the geometric blurring effect of the pinhole.

Another limitation of the pinhole camera technique in this experiment is the choice of a cylindrical cell. The gauge volume defined by the pinhole and incident X-ray beam is shown schematically in Fig. S1(*a*) in the supporting information, which shows the potential for overlap of diffraction signal from different regions of the cell. The implications of this overlap are discussed in the *Results and discussion* section (Section 3). The pinhole camera setup may be better suited to a flat cell with a lamellar structure [Fig. S1(*b*)], with a high-aspect-ratio ‘letterbox’ incident X-ray beam selected so that the pinhole camera only sees an illuminated slice through the battery. A series of 2D slices could then be imaged by translating the cell through the beam.

Further limitations were found with the twin-detector setup. It proved extremely challenging to align both detectors and pinholes on the same region of interest in the cylindrical AA cell. While data were collected using both detectors during the experiment, we only present and discuss the results from one detector.

Finally, a spectroscopic detector like the HEXITEC will pick up background air scattering, so additional shielding, particularly lead-shielded flight tubes around the incident beam, and between the pinhole and detector, was necessary to minimize background counts.

#### 2.2.4. Battery setup and discharge during diffraction measurements   

For the first *operando* discharge experiment, an AA battery was mounted in a custom-made holder mounted on a rotation stage. The battery was discharged through a 2 Ω resistor, resulting in a fast discharge to depletion in approximately 4 h. The cut-off point for the end of the experiment was a remaining battery voltage of 0.8 V, representing 90–95% depth of discharge. The battery voltage and circuit current were continuously logged throughout the discharge process. During discharge, diffraction measurements were made, collecting for 5 min with a 15 min gap between collections, giving a snapshot of the state of the battery every 20 min. For comparison with time-resolved EDXD (Gallaway *et al.*, 2014 ▸), with a 50 µm EDXD gauge volume, 20 s acquisition time and 50 collection points, the total time per map was 50 × 20 s = 1000 s (16.7 min). In the work of Gallaway *et al.* (2015 ▸), again with a 50 µm EDXD gauge volume, 20 s acquisition time and 22 collection points, a spatial map took 22 × 20 s = 440 s or 7.3 min. Note that, for the HEXITEC, the equivalent number of collection points is 80 in 5 min, based on the number of pixel rows or columns, and the data collection per point is in parallel, not sequential.

To improve powder averaging of the diffraction information, the battery sample was rocked around its longitudinal axis by ±25°. The cylindrical symmetry of an AA cell was suitable for this. The rocking of the battery meant that the data were spatially averaged in the circumferential direction while still preserving spatial resolution in the axial direction. Resolution in the radial direction was compromised by the shape of the gauge volume, as discussed in Section 2.2.3.

A second *operando* experiment was performed on a fresh battery, again with discharge through a 2 Ω resistor, but this time discharging the battery for 1 h, followed by 2 h of recovery with the battery disconnected. The discharge process was continued in this manner (1 h on, 2 h off) until the battery was depleted, indicated by a remaining voltage of 0.8 V. As for the first experiment, the acquisition time was 5 min with a 15 min gap between collections, giving a snapshot of the state of the battery every 20 min. This experiment was performed to compare any chemical or microstructural changes taking place in ‘realistic’ use conditions, whereby the battery was used for a period of time and then left at open-circuit voltage for a period until required again.

### 2.3. X-ray tomography   

Tomography on AA cells from the same batch used for the diffraction studies was performed on the I12-JEEP Beamline at Diamond Light Source (Drakopoulos *et al.*, 2015 ▸). The batteries were mounted in the same sample holder and were discharged through a 2 Ω resistor while monitoring battery current and voltage. The battery diameter fitted entirely within the field of view of the X-ray imaging camera, so conventional acquisition and reconstruction procedures were used. The battery was positioned vertically so that the tomography scans included the same region observed for the equivalent cells used in the HEXITEC pinhole diffraction setup. The relevant region of interest was then extracted from the reconstructed tomography data sets for presentation with the diffraction results in the *Results and discussion* section (Section 3). Tomography data sets were acquired at 5–10 min intervals using 75 keV X-rays. For each tomography scan, 1800 projections (0 to 180° with 0.1° step) were recorded with an exposure time of 2.5 ms. The pixel resolution was 7.91 µm per pixel, with a field of view (H × V) of 2560 × 2160 pixels or 22.2 × 17.1 mm. Tomographic reconstruction was performed using the *SAVU* system (Atwood *et al.*, 2015 ▸; Wadeson & Basham, 2016 ▸). A filtered back projection algorithm was used (Ramachandran & Lakshminarayanan, 1971 ▸), as implemented in the *ASTRA* toolbox (van Aarle *et al.*, 2016 ▸). *ImageJ* (Schneider *et al.*, 2012 ▸; Rasband, 1997–2018 ▸) was used for local thresholding, image contrast enhancement, medial filtering, segmentation and analysis of reconstructed images. Differences in the X-ray attenuation between the battery casing, MnO_2_, Zn, ZnO and central current collector resulted in different grey levels in the reconstructed images. Segmentation of different battery regions, shown schematically in Fig. S2, enabled estimation of their macroscopic volume changes based on changes in cross-sectional area. In a cylindrical object of constant length like an AA cell, changes in cross-sectional area are equivalent to changes in volume.

## 3. Results and discussion   

### 3.1. 2 Ω continuous discharge   

In this study, we evaluated the changes in the cathode and anode of a Zn/MnO_2_ battery using *operando* synchrotron tomography and *operando* spatially resolved synchrotron diffraction with one HEXITEC detector. The process investigated was continuous discharge of the battery through a 2 Ω resistor over a period of approximately 4 h. Note that the tomography and diffraction experiments were performed in separate experiments on different batteries from the same batch, with the aim of generating complementary information.

**Figure 3:**
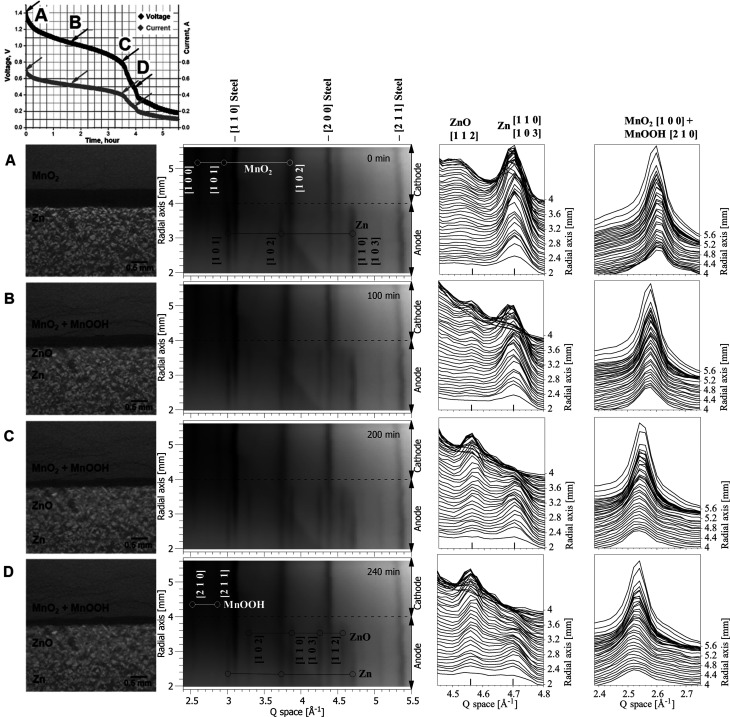
Results for 2 Ω continuous discharge; each row (A, B, C or D) represents a reconstructed tomography slice through the region of interest and the corresponding spatially resolved diffraction patterns at different radial distances from the current collector at one time point on the discharge curve at the top of the image. Stack plots show the transformation of Zn (overlapping peaks 110 and 103) to ZnO (peak 112) in the anode (third column) and the progression of the MnO_2_ (peak 100) to MnOOH (peak 210) reaction in the cathode (fourth column). The cathode reaction is observed as a peak shift because the materials form a continuous solid solution (Gallaway *et al.*, 2015 ▸).

The 2D region of interest shown in Fig. 1 ▸ was mapped with one HEXITEC detector. The aim was to observe dynamically the changes in the spatial distribution of chemical species Zn, ZnO and MnO_2_ at different distances from the separator. An overview of the time-resolved results is presented in Fig. 3 ▸. Complementary tomography measurements on an identical battery provided direct information about the morphological changes in the battery (Fig. S2), allowing correlation of these changes with chemical changes observed in the diffraction measurements.

The diffraction data indicate that, before the start of battery discharge (Fig. 3 ▸, first row), the anode material consisted of Zn and the cathode material consisted of MnO_2_. A reconstructed tomography image of the battery confirms that the distribution of Zn grains in the anode was homogeneous and the MnO_2_ cathode was uniform in morphology.

During discharge, significant morphological changes were observed in the tomographic images (Fig. 3 ▸, first column), which include a macroscopic volume expansion (14%) of the cathode. Multiple cracks appeared in the cathode material, accompanying the transformation of MnO_2_ to MnOOH. Considerable shrinkage of the separator (approximately 3.3 times in volume) and macroscopic contraction of the anode material were observed during the transformation of Zn to ZnO (see Fig. S2 for detail), being in agreement with previously published results (Haibel *et al.*, 2010 ▸; Osenberg *et al.*, 2018 ▸). The calculated macroscopic reduction in anode volume was 6%. In the reconstructed tomographic images, the size of zinc grains measured in 2D slices near the separator decreased from an average size of 150 µm down to an average size of approximately 100 µm at the end of discharge. It was also observed that the zinc particles became less well defined (Fig. S2), and we attribute this to the formation of ZnO around the zinc particles as they reacted (Bhadra *et al.*, 2015 ▸). The morphological change in the anode due to oxidation of Zn particles started next to the separator but did not appear to proceed more than half of the distance between the separator and current collector by the time the battery was discharged. Similar spatial changes in the anode of alkaline cells have been reported elsewhere (Haibel *et al.*, 2010 ▸), with zinc dissolution and ZnO formation occurring preferentially at the separator interface. The morphological changes we observed in the cathode and anode support recent *in situ* tomography results for rechargeable alkaline–manganese batteries (Haibel *et al.*, 2010 ▸; Arlt *et al.*, 2014 ▸; Osenberg *et al.*, 2018 ▸).

The morphological changes in the anode correlate with the spatially resolved diffraction data from an identical battery studied with the HEXITEC detector (Fig. 3 ▸, second and third columns). There is a clear sequential transformation of Zn to ZnO in the direction from the separator towards the current collector. Each row in the diffraction plot corresponds to a radial distance of 46 µm in the battery, which is the pixel resolution of the pinhole camera setup. Note that the finite size of the pinhole dominates the PSF of the detector system, degrading the resolution, as discussed earlier in Section 2.2.3. Hence, there will be overlap in signal between detector rows. Another limitation discussed in Section 2.2.3 is the inter­section of the cylindrical cell with the gauge volume defined by the pinhole and incident beam. This explains why, in Fig. 3 ▸, the MnO_2_ 102 peak extends considerably into the anode region. Despite these limitations, temporal and spatial changes were successfully observed. As a function of time, the intensity of the diffraction peak corresponding to Zn decreased, while the intensity of the diffraction peak corresponding to ZnO increased. The chemical transformation started next to the separator and developed in the direction of the current collector, with the ZnO signal decreasing with increasing distance from the separator. These findings agree with the results of Gallaway *et al.* (2014 ▸), who observed that ZnO reflections first appeared at the anode–separator interface. The ZnO signal spread through the anode at lower intensity. This spatial change in ZnO signal correlates with the tomography results shown in Fig. S3. Qualitatively, the Zn particles near the separator become less well defined. We think this is indicative of a mechanism described by Gallaway *et al.* (2014 ▸) and Bhadra *et al.* (2015 ▸), where a ZnO shell forms around the Zn particles as they transform.

Considering the cathode, the transformation of MnO_2_ to MnOOH over time is observed as a shift in the observed peaks. This transformation was described by Gallaway *et al.* (2015 ▸) as a continuous process of formation of a non-stochiometric solid solution of MnO_2_ and MnOOH, because both materials have similar crystal structures. Ghodbane *et al.* (2012 ▸) confirmed by *in situ* high-resolution powder diffraction measurements that MnO_2_ cathodes exhibit contraction/swelling of the crystal structure upon discharge or charge, due to the intercalation or deintercalation of cations. The reported swelling of the crystal structure in the cathode correlates with our tomographic observations of macroscopic swelling of the cathode.

In all the diffraction results, peaks from the steel casing of the battery are present. It was not possible to eliminate these peaks using the pinhole setup, and there is an apparent shift in the steel peak with radial distance due to the cylindrical geometry of the cell.

### 3.2. 2 Ω interrupted discharge   

**Figure 4:**
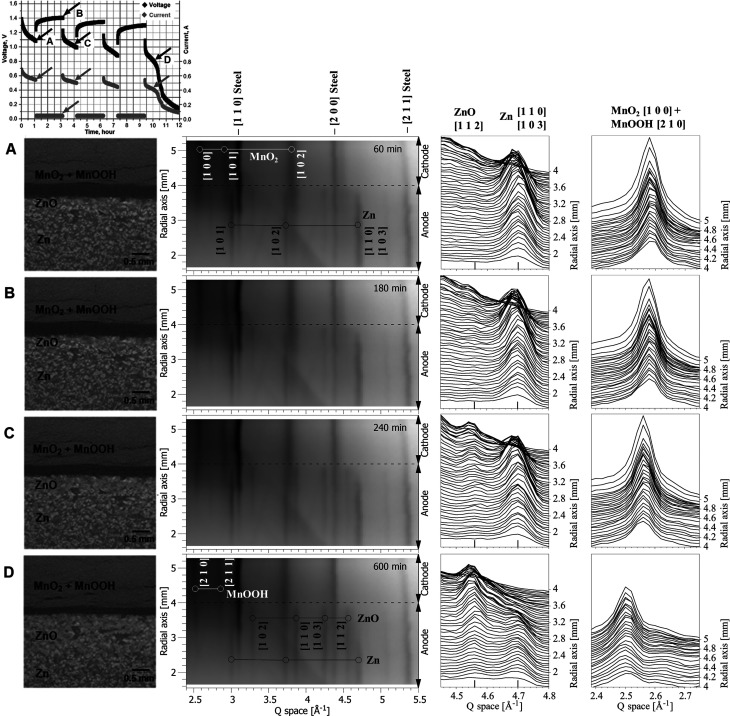
Results for 2 Ω interrupted discharge; each row contains a discharge curve (with the time of the tomography and diffraction measurements indicated by arrows), a reconstructed tomography slice through the region of interest and the corresponding spatially resolved diffraction patterns at different radial distances from the current collector. The third column is for the anode [transformation of Zn (overlapping peaks 110 and 103) to ZnO (peak 112)] and the fourth column is for the cathode [transformation of MnO_2_ (peak 100) to MnOOH (peak 210)]. The cathode reaction is observed as a peak shift because the materials form a continuous solid solution (Gallaway *et al.*, 2015 ▸).

The second investigated discharge process was again through a 2 Ω resistor, but this time the battery was discharged for 1 h followed by a 2 h pause before resuming discharge. The discharge/pause iteration was repeated three times before the battery was completely discharged during the fourth discharge. An overview of the time-resolved results is presented in Fig. 4 ▸. The purpose of the interrupted discharge was to look for signs of changes in battery morphology or chemistry during the pauses in discharge. No morphological or structural changes were observed during the pauses in either the tomography or diffraction data. Overall, the time spent in discharge was 3.5 h, the same as the uninterrupted (continuous) test.

The morphological and chemical changes during interrupted discharge were similar to those observed in the batteries subjected to continuous discharge. The tomography images showed swelling of the cathode, with cracks appearing and compression of the separator. In the anode, the chemical transformation of Zn to ZnO initiated at the separator and developed over time towards the current collector.

**Figure 5:**
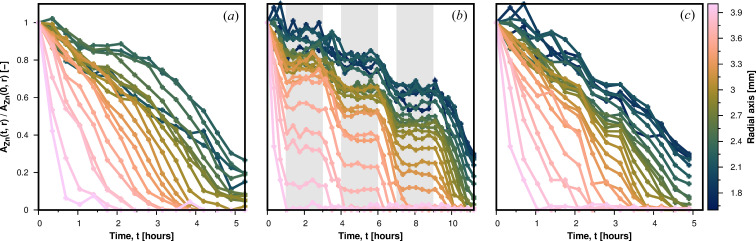
The relative zinc peak area changes *A*_Zn_(*t*, *r*)/*A*_Zn_(0, *r*) as a function of time and position along the radial axis inside the anode. (*a*) During continuous discharge. (*b*) During interrupted discharge. Grey areas mark the pauses in the discharge process. (*c*) Data for interrupted discharge with pause periods not plotted.

**Figure 6:**
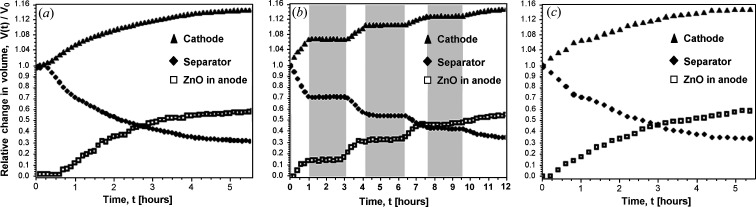
The relative volume change *V*(*t*)/*V*_0_ for cathode, separator and relative volume of ZnO in the anode as a function of time. (*a*) During continuous discharge. (*b*) During interrupted discharge. Grey areas mark the pauses in discharge. (*c*) Data for interrupted discharge plotted without the pause periods.

Further analysis of the spatial and temporal change in anode chemistry is shown in Fig. 5 ▸. Here, the relative changes in the Zn peak area are shown. The Zn peak area *A*_Zn_(*t*, *r*) at time *t* and radial axis position *r* was obtained by fitting the Zn peak (overlapping peaks 110 and 103) from 4.6 to 4.8 Å^−1^ using a Gaussian function. As can be seen from comparison of Figs. 5 ▸(*a*) and 5 ▸(*c*), there is no significant difference in the spatial and temporal change in anode chemistry in the cases of continuous and interrupted discharge. There is, however, a difference between the two batteries, seen as a difference in the relative zinc peak area close to the current collector, near radial distance 1.8 mm. The Zn peak area is higher near the current collector for the battery subjected to interrupted discharge [Fig. 5 ▸(*c*)] compared with the battery that was continuously discharged [Fig. 5 ▸(*a*)]. We think this may be attributed to a variation in the quantity of zinc particles in the gel anode of the two batteries tested, even though they were from the same batch. Further investigation would be required to confirm this. Again comparing continuous and interrupted discharge, there is also no difference in the relative volume change of the cathode and anode and in the amount and spatial distribution of ZnO, as follows from measurements made on the tomography data (Fig. 6 ▸). Thus, there is a clear correlation between structural changes in the anode material measured by energy-dispersive diffraction with the HEXITEC detector and macroscopic changes in the battery components measured by conventional X-ray tomography. Combined measurements using both experimental techniques in one experimental setup would allow simultaneous *operando* measurements of the structural and morphological changes in an intact commercial-sized battery.

## 4. Conclusions   

Using the example of well understood standard battery discharge, we have demonstrated that the combination of *operando* spatially resolved synchrotron diffraction and *operando* synchrotron tomography is a promising method for monitoring chemical and macroscopic changes in complete battery cells. The 2D energy-dispersive diffraction information obtained using an 80 × 80 pixel HEXITEC CdTe detector and pinhole camera removes the requirement to scan samples point by point through a gauge volume (as in conventional EDXD). However, the low efficiency of the pinhole system means that the time resolution is still of the order of minutes. The finite size of the pinhole is a limitation of the technique, causing a degradation in spatial resolution. The geometry of the setup is also not optimal for a cylindrical cell, resulting in spatial overlap of diffraction information. The energy resolution of the HEXITEC CdTe sensor is also lower than that of the high-purity Ge crystals used in energy-dispersive detectors.

*Operando* tomography confirmed the morphological changes in Zn/MnO_2_ alkaline cells observed by others. The macroscopic volume changes of cathode and anode could be tracked and, qualitatively, the transformation of Zn to ZnO in the anode was observed to be strongest near the separator, complementing the observed spatial variation in diffraction signal intensity of Zn and ZnO.

## 5. Further work   

It is possible, with existing equipment, to combine 2D diffraction data collection by the HEXITEC detector with simultaneous white-beam tomographic imaging. As this is a white-beam technique, the experiment could be attempted on a laboratory X-ray setup, although the lower flux will compromise time resolution. A development for the HEXITEC setup that would be a significant improvement over the pinhole camera arrangement would be individual pixel collimation. Production of a high-aspect-ratio pixellated collimator array remains a significant technical challenge. Improvements in detector performance may be achieved with a recently developed CdZnTe sensor for the HEXITEC detector, as well as via increasing the size of the sensor and the frame rate. It is proposed that the pinhole detector setup may be better suited to lamellar batteries illuminated with a high-aspect-ratio incident X-ray beam.

## Supplementary Material

Additional figures. DOI: 10.1107/S1600576720012078/iu5001sup1.pdf

## Figures and Tables

**Table 1:** Geometric information for the HEXITEC pinhole camera setup

Sensor size	20 × 20 mm
Sensor size	80 × 80 pixels
Pixel size	250 µm
Sample-to-pinhole distance	90 mm
Pinhole-to-detector distance	490 mm
Pinhole diameter	200 µm
Magnification	5.4
Field of view at sample	3.7 × 3.7 mm
Resolution	46 µm per pixel
2θ angle	5°
