# Supplementary material for: An operando spatially resolved study of alkaline battery discharge using a novel hyperspectral detector and X-ray tomography
Source: J Appl Crystallogr. 2020 Oct 13;53(Pt 6):1434–43. doi: 10.1107/S1600576720012078 (PMC7710487; doi:10.1107/S1600576720012078)
Supplement: Supplementary file 1 [file j-53-01434-sup1.pdf]

JOURNAL OF APPLIED CRYSTALLOGRAPHY

Volume 53 (2020)

Supporting information for article:

An *in operando* spatially resolved study of alkaline battery discharge using a novel hyperspectral detector and X-ray tomography

Thomas Connolley, Oxana V. Magdysyuk, Stefan Michalik, Phoebe K. Allan, Manuela Klaus, Paul H. Kamm, Francisco Garcia-Moreno, Jennifer A. Nelson, Matthew C. Veale and Matthew D. Wilson

S1. Supplementary Figures

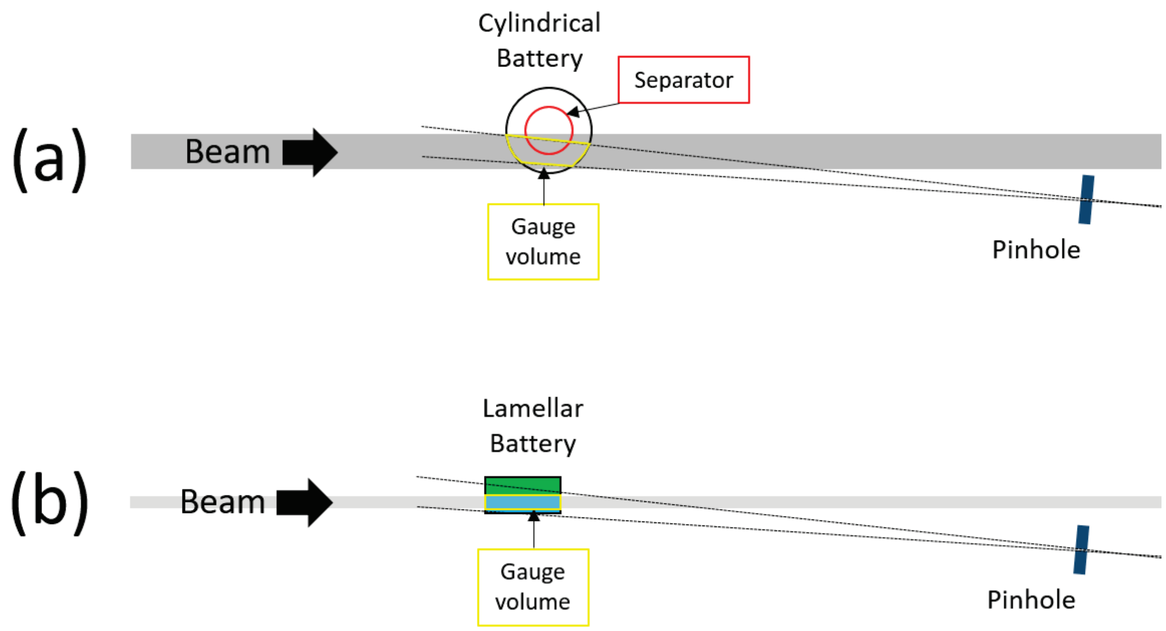

Figure S1 Schematic plan views of gauge volume defined by the pinhole and incident X-ray beam. (a) The potential overlap between different regions of a cylindrical battery cell. (b) Shows how the pinhole imaging technique may be better suited to a lamellar battery geometry.

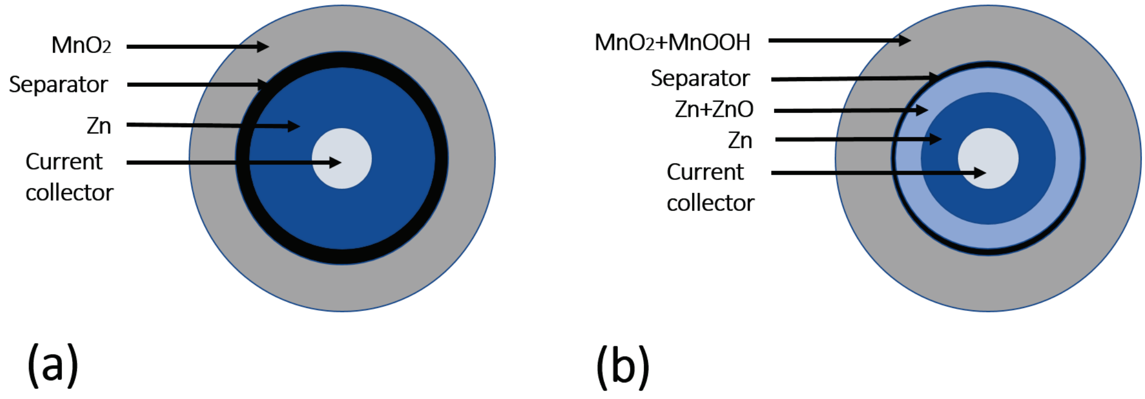

Figure S2 Schematic showing the segmentation of tomographic slices through the AA cell. (a) Before discharge. (b) After discharge.

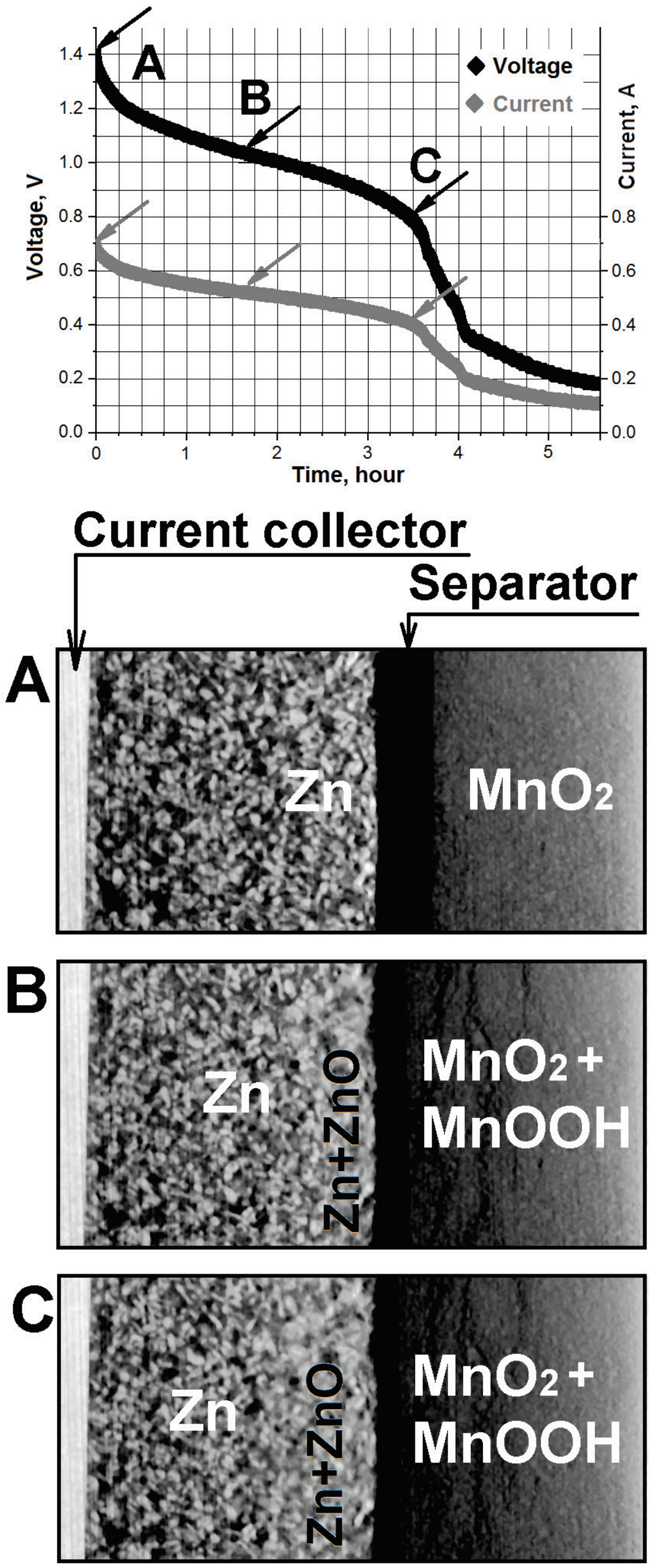

Figure S3 Detail from vertical section through the tomographic reconstruction of the cell subjected to 2 ohm Continuous Discharge; A, B, and C show the same region in the battery at different states of discharge, showing morphological changes in the cathode, separator and anode.
